# Why do different oceanic archipelagos harbour contrasting levels of species diversity? The macaronesian endemic genus *Pericallis* (Asteraceae) provides insight into explaining the ‘Azores diversity Enigma’

**DOI:** 10.1186/s12862-016-0766-1

**Published:** 2016-10-08

**Authors:** K. E. Jones, S. Pérez-Espona, J. A. Reyes-Betancort, D. Pattinson, J. Caujapé-Castells, S. J. Hiscock, M. A. Carine

**Affiliations:** 1 Botanischer Garten und Botanisches Museum Berlin-Dahlem, Dahlem Centre of Plant Sciences, Freie Universität Berlin, Königin-Luise Str. 6-8, Berlin, 14195 Germany; 2 Estación Biológica de Doñana, CSIC, C./ Américo Vespucio s/n, Sevilla, E-41092 Spain; 3 Jardín de Aclimatación de La Oratava (ICIA), C/Retama 2, Puerto de la Cruz, Tenerife 38400 Spain; 4 Natural History Museum, Cromwell Road, London, SE7 5ED UK; 5 Present address: Department of Zoology, University of Cambridge, Downing Street, Cambridge, CB2 3EJ UK; 6 Jardín Botánico Canario “Viera y Clavijo”-Unidad Asociada al CSIC (Cabildo de Gran Canaria), Camino del palmeral 15 (Tafira Alta), Las Palmas de Gran Canaria, 35017 Spain; 7 Department of Plant Sciences, University of Oxford, South Parks Road, Oxford, OX1 3RB UK

**Keywords:** Ecological variation, Genetic diversity, Macaronesia, Morphological diversity, *Pericallis*, Population genetics

## Abstract

**Background:**

Oceanic archipelagos typically harbour extensive radiations of flowering plants and a high proportion of endemics, many of which are restricted to a single island (Single Island Endemics; SIEs). The Azores represents an anomaly as overall levels of endemism are low; there are few SIEs and few documented cases of intra-archipelago radiations. The distinctiveness of the flora was first recognized by Darwin and has been referred to as the ‘Azores Diversity Enigma’ (ADE). Diversity patterns in the Macaronesian endemic genus *Pericallis* (Asteraceae) exemplify the ADE. In this study we used morphometric, Amplified Length Polymorphisms, and bioclimatic data for herbaceous *Pericallis* lineages endemic to the Azores and the Canaries, to test two key hypotheses proposed to explain the ADE: i) that it is a taxonomic artefact or Linnean shortfall, ie. the under description of taxa in the Azores or the over-splitting of taxa in the Canaries and (ii) that it reflects the greater ecological homogeneity of the Azores, which results in limited opportunity for ecological diversification compared to the Canaries.

**Results:**

In both the Azores and the Canaries, morphological patterns were generally consistent with current taxonomic classifications. However, the AFLP data showed no genetic differentiation between the two currently recognized Azorean subspecies that are ecologically differentiated. Instead, genetic diversity in the Azores was structured geographically across the archipelago. In contrast, in the Canaries genetic differentiation was mostly consistent with morphology and current taxonomic treatments. Both Azorean and Canarian lineages exhibited ecological differentiation between currently recognized taxa.

**Conclusions:**

Neither a Linnean shortfall nor the perceived ecological homogeneity of the Azores fully explained the ADE-like pattern observed in *Pericallis*. Whilst variation in genetic data and morphological data in the Canaries were largely congruent, this was not the case in the Azores, where genetic patterns reflected inter-island geographical isolation, and morphology reflected intra-island bioclimatic variation. The combined effects of differences in (i) the extent of geographical isolation, (ii) population sizes and (iii) geographical occupancy of bioclimatic niche space, coupled with the morphological plasticity of *Pericallis*, may all have contributed to generating the contrasting patterns observed in the archipelagos.

**Electronic supplementary material:**

The online version of this article (doi:10.1186/s12862-016-0766-1) contains supplementary material, which is available to authorized users.

## Background

A key question for biologists is: why do different geographic regions harbour contrasting levels of biodiversity? [1–4]. Oceanic archipelago floras provide striking examples of flowering plant lineages that have undergone extensive adaptive and allopatric diversification, with a high proportion of Single Island Endemics (hereafter SIEs) for example, the Lobelioids in the Hawaiian archipelago [5] and the *Aeonium* alliance in the Canary Islands [6]. However, the Azores archipelago, part of the Macaronesian region *sensu* Dansereau [7] that also comprises the Cape Verde, Canaries, Salvagems and Madeira, represents an anomaly and shows a much lower proportion of SIEs when compared to other archipelagos [8]. Furthermore, in the Azores there are few examples of taxa that have diverged in situ, with 80 % of endemic lineages containing just a single endemic taxon; in the Canaries, this figure is 56 % [8–10]. This phenomenon was first alluded to by Darwin in a letter to Joseph Hooker dated Christmas Day, 1844, where he commented on a recently published enumeration of the Azores flora [11] and noted: “*Watson’s paper on [the] Azores has surprised me much; do you not think it odd, the fewness of peculiar species…?*” [12]. Carine and Schaefer [8] coined the term the ‘Azores Diversity Enigma’ (ADE henceforth) to collectively refer to these two distinctive features of the Azores flora, i.e. the limited incidence of evolutionary radiations and paucity of SIEs in the flora.

Hypotheses to explain the ADE have included the proposal that the Azorean islands, or the lineages inhabiting them, are too young for extensive radiations to have occurred - with ca. 62 % of the land area being less than 1 million years old [13–15], that they are too small in land surface area [15] or that, in contrast to other archipelagos, the Azorean islands are too ecologically homogeneous to have facilitated extensive diversification [15, 16]. Carine and Schaefer [8] suggested that these hypotheses do not satisfactorily explain the distinctive patterns in the Azores, highlighting potential influence of inconsistent taxonomic effort or different palaeo-climate conditions on the evolution of their floras. Schaefer et al. [10] subsequently investigated genetic diversity patterns in ca. 20 % of Azorean endemic lineages using the Internal Transcribed Spacer region of nuclear ribosomal DNA (ITS) sequences and found higher levels of molecular diversity and molecular SIEs compared to current taxonomic concepts. The authors concluded that the ADE could indeed be a taxonomic artefact (Linnean shortfall).

**Fig. 1:**
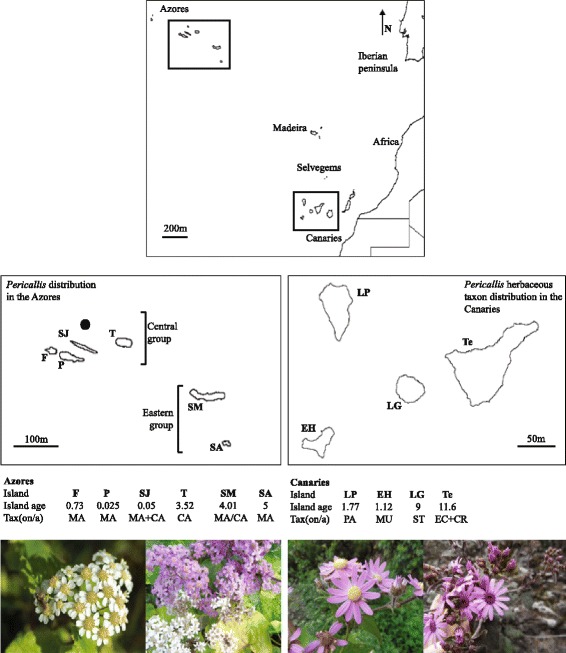
Geographic setting of the study archipelagos: Azores and Canaries in Macaronesia (above) and islands of occupancy of *Pericallis* lineages used in this study. Islands: F, Faial; P, Pico; SJ, São Jorge; T, Terceira; SM, São Miguel; SA, Santa Maria; LP, La Palma; EH, El Hierro; LG, La Gomera; Te, Tenerife. Island ages are maximum ages in Myr taken from Caujapé-Castells (2010). Taxa: MA, *P. malvifolia* subsp*. malvifolia*; CA, *P. malvifolia* subsp. *caldeirae*; PA, *P. papyracea*; MU, *P. murrayi*; ST, *P. steetzii*; EC, *P. echinata*; CR, *P. cruenta*. A black circle is used to indicate the presence of the island Graciosa, which does not host any populations of *P. malvifolia* and, therefore, was not sampled in this analysis. Photos (left to right): *P. malvifolia* subsp*. caldeirae*, Azores, São Miguel, Lagoa do Fogo, photo credit: H. Schaefer; *P. malvifolia* subsp*. malvifolia*, Azores, São Miguel, Madrugada, collection: Jones et al. 282, photo credit: José Martins; *P. echinata*, Canaries, Tenerife, Teno, collection: Jones et al. 195, photo credit: KE Jones; *P. cruenta*, Canaries, Tenerife, La Orotava, collection: Jones et al. 243, photo credit: KE Jones

The genus *Pericallis* (Senecioneae, Asteraceae) is endemic to Macaronesia. With sixteen species and a distribution spanning the Azores, Canaries and Madeira, it exemplifies the ADE, since diversity in this genus is unevenly distributed across the region: 14 taxa occur in the Canaries, 11 of which are SIEs, two SIEs occur in the Madeira archipelago [17] and one species with two multi-island endemic (MIE) subspecies occurs in the Azores (Fig. 1).

Jones et al. [18] identified two herbaceous lineages, namely the Azorean lineage and a lineage comprising five SIE species in the Canaries that diverged recently (ca. 0.89 Ma (0.09–2.9 Highest Posterior Density (HPD)) and ca. 1.32 Ma (0.007–3.24 HPD) respectively). Despite being of a similar age, the Azorean and Canarian lineages exhibit marked differences in their diversity patterns. The five SIEs of the Canarian lineage (*P. cruenta, P. papyracea, P. murrayi, P. steetzii* and *P. echinata*) all exhibit broad and overlapping altitudinal and habitat ranges (Fig. 1; see [18]). In contrast, the two Azorean endemic taxa are ecologically differentiated MIEs with overlapping island distributions: *P. malvifolia* subsp. *malvifolia* is restricted to low altitudes (<300 m) on Santa Maria, São Miguel, Pico, Faial and São Jorge; *P. malvifolia* subsp. *caldeirae* is restricted to higher altitudes (>500 m) and is found on São Miguel, Faial, Terceira and Pico [19]; see Fig. 1. The Azorean and Canarian groups thus exhibit markedly different patterns that reflect the ADE.

Phylogenetic analyses of chloroplast and nuclear ribosomal ITS data provided only limited resolution within these clades but they did not support current taxonomic treatments in the Azorean clade [18]. The two ecologically distinct Azorean subspecies were not distinguished and the data were rather consistent with a pattern of geographic structuring across the Azores. In the Canarian lineage, little genetic differentiation was observed between the five currently recognized taxa with sharing of haplotypes evident between some taxa according to some of the markers used. Whilst high morphological divergence with low sequence diversity is common in island radiations [20], morphology-based species delimitation in the Canarian lineage has been called into question with both Nordenstam [21] and Swenson and Manns [22] suggesting that taxonomic concepts for *Pericallis* need to be re-assessed.

The goal of this study is to understand the contrasting diversity patterns observed in the Azores and Canaries, focussing specifically on herbaceous *Pericallis* lineages endemic to each archipelago. Using morphology, Amplified Fragment Length Polymorphisms (AFLPs) and bioclimate data we test two hypotheses to explain the ADE (Table 1): (i) that differences are the result of taxonomic artefact or Linnean shortfall resulting from the under description of taxa in the Azores or the over-splitting of taxa in the Canaries [8] and (ii) that differences are related to the greater ecological homogeneity of the Azores [15]. We specifically assess patterns of morphological and molecular variation in the Azorean and Canarian herbaceous lineages, and investigate the relationship between morphological and molecular patterns of variation and geographical and ecological distance.

**Table 1:** Hypotheses to explain the Azores Diversity Enigma

Hypotheses	Justification
1. Linnean shortfall	The differences in diversity patterns of *Pericallis* between the Azores and the Canaries are explained by differences in taxon concepts and/or taxonomic effort applied between the archipelagos. Recent studies have shown that there is potentially greater diversity in the Azores compared to current species circumscriptions [10, 18, 30]
2. Ecological homogeneity	Adaptive diversification plays a key role in the evolution of island lineages [43]. There is more limited opportunity for diversification in the Azores because they are more ecologically homogenous, compared to the Canaries [15].

## Results

### Morphological variation

**Fig. 2:**
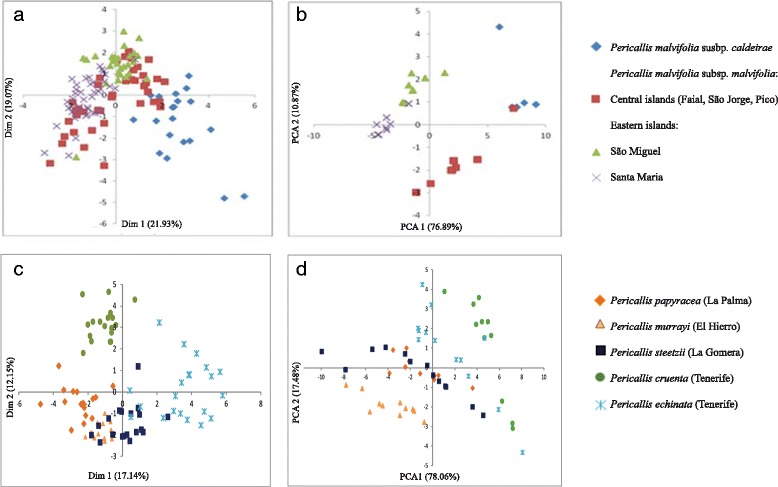
Factor Analyses of Mixed Data of morphological variation (**a**, **c**), and Principal Component Analyses of 19 bioclimatic variables for collection points (**b**, **d**) in each archipelago; Azores (**a**, **b**) and Canaries (**c**, **d**). Each point represents an individual

In both the Azores and Canaries morphological analyses were generally consistent with current taxonomic classifications (Fig. 2a, d). In the Azores, Factor Analysis for Mixed Data (FAMD) of twelve variable morphological characters and 125 individuals revealed the separation of the two subspecies across dimensions one and two, although some overlap between ssp. *malvifolia* from the central islands and ssp. *caldeirae* was evident (Fig. 2a; see Additional file 1: Figure S1 for FAMD plots with points coloured by islands). The first dimension described 21.93 % of the variation and the characters that contributed most significantly to this dimension (with a factor loading > 0.3) were the length of the highest bract and the indumentum of the disc cypselae (Additional file 4: Tables S1 and Additional file 5: Table S2 for morphological data and factor loadings, respectively). The second described 19.07 % of the variation; the most significant character contributing to this dimension was the length of the disc floret corolla. There were also trends suggesting some geographic structuring within *P. malvifolia* subsp. *malvifolia*, since individuals from Santa Maria and São Miguel showed some separation along the second dimension (Fig. 2a). However, individuals from the central islands overlapped with both Santa Maria and São Miguel individuals. Subsequent dimensions provided no useful information regarding differences among the populations investigated.

In the Canaries, the results of the FAMD analysis of 23 variable morphological characters and 89 individuals broadly agreed with current taxonomic treatments, although there was considerable overlap of *P. steetzii* and *P. murrayi* individuals and some overlap of *P. echinata* and *P. steetzii* accessions (Fig. 2c). The first dimension described 17.14 % of the variation and mainly separated *P. echinata* from all other taxa. The characters that contributed most to this dimension are related to capitulum size and length of scales on the phyllary bracts: *P. echinata* exhibits longer disc and ray floret corolla tubes with longer and more abundant scales on the phyllary bracts compared to all other taxa. *Pericallis papyraceus* was also separated from all other taxa along the first dimension; the character that distinguished it along this dimension was the smaller capitulum width. *Pericallis cruenta* was separated from *P. papyraceus, P. steetzii* and *P. murrayi* along the second dimension, which described 12.15 % of the variation (Fig. 2c). The character that contributed most significantly to this dimension and separated *P. cruenta* from all other taxa was abaxial leaf indumentum colour: *P. cruenta* typically exhibits purple abaxial leaf indumentum, whereas *P. papyraceus, P. steetzii* and *P. murrayi* are green to white. With the exception of a strong contribution of the number of the ray florets to the differentiation of *P. papyraceus* (7–8 ray florets) from all other taxa (>10 florets), subsequent dimensions provided no useful information regarding the differences amongst the populations investigated. Non-parametric permutational multivariate analyses of variance (perMANOVA) of morphological data in the Azores revealed a significant difference between taxa and islands (*P* = 0.001). R^2^ values were 0.19 and 0.35 for the analyses of variation between species and between islands, respectively (Table 2). perMANOVA of *Pericallis* morphological data in the Canaries revealed a significant difference between taxa and islands (*P* = 0.001). R^2^ values were 0.65 and 0.43 for analyses between species and between islands respectively (Table 2).

**Table 2:** Results of permutational analysis of variance of *Pericallis* morphological data to assess significant differences between islands and taxa in the Azores and Canaries

Archipelago	Grouping	d.f.	R^2^	*P*-value
Azores	Taxa	1	0.19	0.001
	Islands	5	0.35	0.001
Canaries	Taxa	4	0.65	0.001
	Islands	3	0.43	0.001

### Bioclimatic variation

Nineteen bioclimatic variables from the geographic locations of 125 individuals in the Azores available through Worldclim (http://www.worldclim.org/) were analysed using Principal Component Analysis (PCA). A number of clusters were separated along the first and second axis (Fig. 2b). These corresponded to (i) Santa Maria subsp. *malvifolia* (ii) São Miguel subsp. *malvifolia* (iii) central sub-archipelago subsp. *malvifolia,* (iv) São Miguel subsp. *caldeirae* and (v) central sub-archipelago subsp. *caldeirae* (including one accession of subsp. *malvifolia* from Pico). PC1 explained a much higher percentage of the variation than PC2 (76.89 % vs. 10.87 %, respectively). The most significant ecological variables that contributed to PC1 were all related to precipitation (precipitation of the warmest quarter, wettest quarter and wettest month); those contributing to PC2 were precipitation of the driest quarter and the driest month and isothermality (annual mean temperature range/mean diurnal range: a measure of temperature “evenness” throughout the year; Additional file 5: Table S5).

The PCA of bioclimatic variation in the Canaries for 89 georeferenced individuals showed some separation of the currently recognized taxa: *P. cruenta* and *P. murrayi* were distinguished, although *P. echinata* individuals overlapped with some individuals of *P. cruenta*. Significant overlap was found between *P. steetzii* and *P. murrayi* accessions (Fig. 2d). The most significant ecological variables that contributed to PC1, which explained 78.06 % of the variation were all related to temperature (mean temperature of the coldest quarter, annual mean temperature and maximum temperature of the warmest month); those contributing to PC2 (17.48 %) were isothermality, mean diurnal range and annual temperature range (Additional file 6: Table S3).

### Spatial structuring of genetic variation

**Fig. 3:**
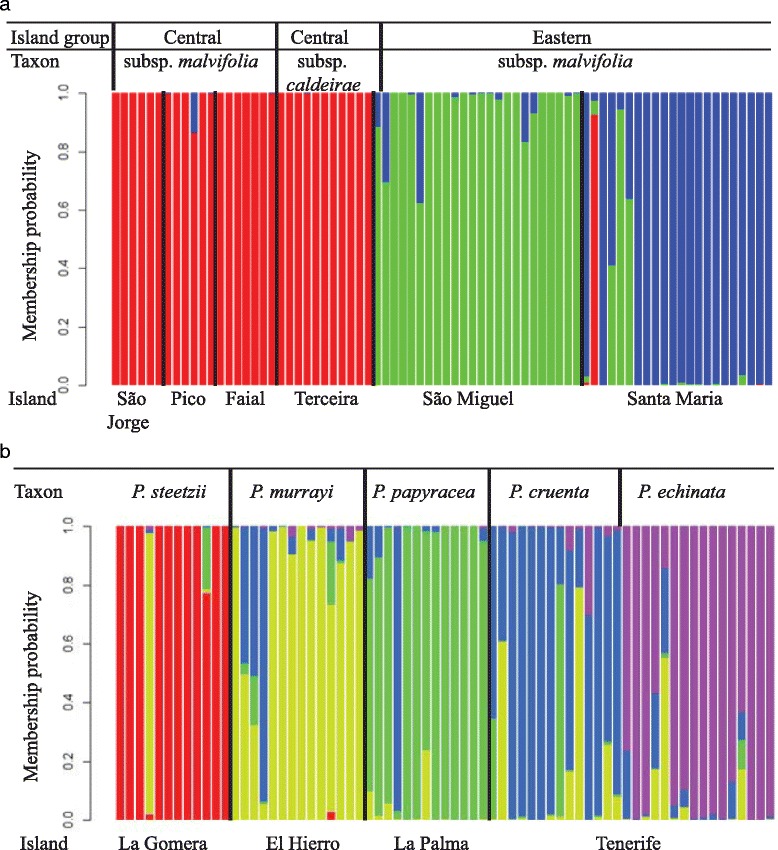
Discriminant Analysis of Principal Components showing the genetic clustering of populations of *Pericallis* lineages analysed in the Azores and Canaries based on AFLP data. Each bar represents one individual plant. (**a**) 76 individuals from the Azores (*K* = 3). *Pericallis malvifolia* subsp. *malvifolia* is separated into different island groupings: Central island subsp. *malvifolia* and Eastern island subsp. *malvifolia*. Taxon names and island groupings are indicated above the plot. Island names are indicated below the plot and separated by bold lines. (**b**) 69 individuals from the Canaries (*K* = 5). Taxon names are indicated above the plots and island names are indicated below the plot separated by bold lines

Seventy-six samples of the Azorean *P. malvifolia* were used for AFLP fingerprinting analysis, 51 of which were also used in the morphometric analysis. This sampling encompassed populations of both subspecies on all islands where they occur. A Discriminant Analysis of Principal Components (DAPC) was used to assign individuals to genetic clusters [23, 24] with selection of the optimal number of clusters based on the Bayesian Information Criterion (BIC; [24]). The results of the *K*-means clustering analyses of the Azorean AFLP data suggested that the best *K* value was 3 (Additional file 2: Figure S2). No genetic differentiation between *P. malvifolia* subsp. *malvifolia* and subsp. *caldeirae* was apparent (Fig. 3a). Rather, there was geographical structuring with the three groups largely restricted to the central sub-archipelago, São Miguel, and Santa Maria, respectively; albeit with some genetic material shared between the groups. Hierarchical analyses of molecular variance (AMOVA) [25, 26] were used to investigate partitioning of variation within and among the groups defined by the DAPC analysis. The AMOVA results suggested that there was greater variation within than between groups (Table 3).

**Table 3:** Hierarchical partitioning of AFLP variation based on Analyses of Molecular Variance of *Pericallis* species in the Azores and Canaries. *P* < 0.001

Archipelago	Source of variation	d.f.	Percentage of variation
Azores (K = 3)	Among groups	2	21.08
	Among populations within groups	11	0.77
	Within populations	69	78.14
Canaries (K = 5)	Among groups	4	10.37
	Among populations within groups	9	2.34
	Within populations	57	87.28

For the Canarian dataset, 69 samples were used to represent the five taxa recognised. Forty-four of the samples were also used in the morphometric analysis. In the DAPC analysis, the most likely *K* values were *K* = 3–5 (Additional file 2: Figure S2) but the differences between the BIC values for these were marginal. We present the DAPC plot for *K* = 5 to reflect the number of taxa currently recognized and the results from cp and ITS sequence data in Jones et al. (2014b); this reveals a pattern that broadly corresponded to the currently accepted taxa but with more sharing of genetic material between taxa than in the Azorean dataset (Fig. 3). For *K* = 4, geographic structuring between islands was observed and for *K* = 3, very little geographic structure was apparent (Additional file 3: Figure S3). As with the Azorean dataset, AMOVA suggested that there was greater variation within groups defined by the DAPC analysis than between groups but genetic differentiation between groups was lower in the Canaries than for the Azorean data set (Table 3).

In the Azores, a full distance-based redundancy analyses (dbRDA) revealed a significant positive correlation between geographic distance and genetic distance but no significant correlation between geographical and morphological distance (Table 4). There were significant correlations between genetic distance and climate PC1 and PC2. However, partial dbRDA, which allows for the fitting of covariates to account for potential confounding effects of these values [27], suggested that climate PC1 and PC2 were not significant for AFLP data variation in the Azores when geographic distance was taken into account. Morphological distance was not correlated with climate PC1 in the full dbRDA analysis, but was correlated with climate PC2. In the partial analysis where geographic distance was taken into account, significant correlations were observed between morphological variation and both climate PC1 and PC2 (Table 4).

**Table 4:** Relationships between genetic and morphological diversity of *Pericallis* and geographic distance (metres) and PC1 and PC2 of the principle coordinate analysis of bioclimatic data using distance-based redundancy analyses. Left: full tests of individual sets. Right: partial tests

Marginal tests				Partial tests (geographic distance)			
Canaries: Euclidean distance matrix (AFLPs)							
Variable	*F*	*P*	Variance	Variable	*F*	*P*	Variance
Distance	1.14	0.005**					
Climate PC1	0.906	0.009**	0.906	Climate PC1	1.0263	0.385	0.589
Climate PC2	0.711	0.124	0.711	Climate PC2	0.9573	0.544	0.549
Canaries: Morphology distance matrix							
Variable	*F*	*P*	Variance	Variable	*F*	*P*	Variance
Distance	3.234	0.005**					
Climate PC1	6.495	0.002**	0.5239	Climate PC1	1.1738	0.276	0.047
Climate PC2	3.357	0.004**	0.1679	Climate PC2	0.8745	0.544	0.035
Azores: Euclidean distance matrix (AFLPs)							
Variable	*F*	*P*	Variance	Variable	*F*	*P*	Variance
Distance	4.913	0.005**					
Climate PC1	4.886	0.002**	1.5171	Climate PC1	0.339	0.188	1.14
Climate PC2	3.285	0.002**	1.0201	Climate PC2	0.288	0.534	0.97
Azores: Morphology distance matrix							
Variable	*F*	*P*	Variance	Variable	*F*	*P*	Variance
Distance	1.055	0.568					
Climate PC1	0.103	0.094‘	1.7331	Climate PC1	0.1606	0.006**	3.072
Climate PC2	0.161	0.008**	2.7107	Climate PC2	0.1537	0.008**	2.941

Significance: *** 0.001, ** 0.01, * 0.05, ‘0.1

In the Canaries, full dbRDA revealed significant positive correlations between both geographic distance and genetic distance, and geographical distance and morphological distance (Table 4). Full dbRDA suggested significant correlations between genetic distance and climate PC1. A significant correlation between morphological distance and climate PC1 and climate PC2 was also observed. However, when geographic distance was taken into account in the partial dbRDA, no significant correlations between genetic distance and morphological or bioclimatic variables were observed.

## Discussion

The low numbers of SIEs in the Azores relative to other oceanic islands was noted by Darwin [12], and was recently termed the ‘Azores Diversity Enigma’ [8]. We aimed to test hypotheses that explain the ADE by comparing patterns of endemicity observed in Azorean and Canarian *Pericallis* lineages that are endemic to each archipelago. We specifically tested two hypotheses (Table 1): (i) the Linnean shortfall hypothesis [8, 28] and (ii) the environmental homogeneity hypothesis [15].

### Hypothesis 1: linnean shortfall in the Azores

The Linnean shortfall hypothesis – the failure to recognise morphologically differentiated taxa in the Azores or over-splitting of taxa in the Canaries – does not adequately explain the ADE for *Pericallis*, contrary to the suggestion of Schaefer et al. [10] and Carine et al. [8] that this may explain the distinctive patterns in the Azores flora more generally. Differences between taxa significantly explained morphological variation of *Pericallis* in the Canaries (65 % of the variation) with species circumscriptions in the Canaries also broadly supported by FAMD analysis of morphological variation. However, *P. steetzii* and *P. murrayi* were not differentiated in the FAMD analysis, which suggests some level of over-splitting (Fig. 2c) but not enough to adequately explain the ADE. We also observed a significant correlation between morphological and geographical distance, with differences between islands explaining 43 % of the morphological variation. This is consistent with the recognition of SIEs, even though considerable morphological variation exists within islands, as is evident from the space occupied by taxa in the FAMD analysis (Fig. 2c). Similarly, the FAMD analysis of morphological variation in the Azores broadly supported current taxon delimitation, largely differentiating between the low altitude subsp. *malvifolia* and the high altitude subsp. *caldeirae* (Fig. 2a). The perMANOVA analysis revealed that morphological variation was better explained by differences between islands (35 %) than by differences between subspecies (19 %). However, there are two morphological characters that are markedly distinct between subspecies, namely disc cypselae indumentum and length of the highest bract. Populations of subsp. *malvifolia* from Santa Maria and São Miguel show some morphological differentiation according to the FAMD that is not reflected in current treatments (Fig. 2a: see supporting information S4 for FAMD plots with points coloured by islands) and that largely reflects differences in disc floret corolla length and stamen length. However, this variation is subsumed within the range of morphological variation exhibited by the central group subsp. *malvifolia*, which precludes its taxonomic recognition. The larger morphological variation found between islands in the Azores according to perMANOVA may reflect the combined effect of 1. the morphological differences between Santa Maria and São Miguel and 2. the morphological differences between subspecies *caldeirae* restricted to the central islands and subsp. *malvifolia* that occurs on both central and Eastern island groups.

In contrast to the situation in the Canaries, there was no correlation between morphological and geographical distance in the Azores (Table 4). This is in contrast to the situation in some other Azorean plant groups, where recent taxonomic revision has resulted in the recognition of geographically restricted endemic taxa (e.g. *Platanthera,* Bateman [29]; *Leontodon* [30]; *Aichryson* [31]). Analyses of patterns of morphological variation in *Pericallis* therefore suggest that past failures to recognise morphologically distinct taxa in the Azores do not appear to be an explanation for the lack of SIEs in Azorean *Pericallis* even though it may be significant in other groups.

### Hypothesis 2: ecological homogeneity in the Azores

Ecology would appear to be an important factor associated with diversification in both archipelagos. Both Azorean and Canarian lineages exhibited ecological differentiation between currently recognized taxa, although differentiation was greater between the Azorean taxa (Figs. 2b and d). Furthermore, variation in morphology was correlated with climate and geographical distance in both the Canaries and the Azores (Table 4). In the Canarian lineage, species exhibited broad ecological ranges but ecological differentiation between species is nevertheless observed in the PCA analysis (Fig. 2d). Morphology was correlated with both PC1 and PC2 of the climate analysis, although this result was highly influenced by the effect of geographical distance (Table 4). The Azorean pattern of climatic differentiation was similarly consistent with morphological differentiation, yet with greater clustering in the bioclimatic PCA compared to the FAMD based on morphological data (Fig. 2a and b). It is notable that most of the variation in climate (76.89 %) was explained by the first axis of the PCA analysis. Along axis PC1, all subsp. *caldeirae* individuals, with the exception of one individual from São Miguel were differentiated from subsp. *malvifolia*. Populations of subsp. *malvifolia* from Santa Maria, São Miguel and the central group were also differentiated in the PCA of climatic data. Whilst morphology showed no correlation with geographical distance, it was correlated with climate PC2, and strongly so according to the partial dbRDA when geographical distance was taken into account (Table 4). The results therefore suggest that shifts in bioclimatic preference across an ecologically heterogeneous island system are associated with the morphological diversification of *Pericallis* groups in both the Azores and the Canaries. In both archipelagos, there are more floral than leaf characteristics accounting for the morphological differentiation between the bio-climatically distinct *Pericallis* taxa, for example, cypselae indumentum of the disc florets between the ecologically and attitudinally distinct *P. malvifolia* subspecies in the Azores. These traits are not obviously adaptive, a situation in contrast to some other island radiations studied wherein variation has been observed in leaf characters, for example, *Plantago* in Hawaii [32] and *Lavandula* in the Canary Islands [33]. The WorldClim model interpolates from weather station observations using latitude, longitude and elevation. Therefore, there may be limitations in the reliability of the bio-climatic data, particularly in regions with varied topography such as oceanic archipelagos [34, 35]. Despite this, we identify clear bioclimatic patterns in the case of *Pericallis* in the Azores and Canaries at odds with the ecological homogeneity hypothesis [14]. In order to further test the ecological homogeneity hypothesis put forward by Triantis et al. [14], it would be informative to measure and compare the levels of ecological opportunity between taxa in the Azores and Canaries and test the associations with adaptive radiations. This would, for example, require an assessment of potential key innovations and the colonization of new habitats and subsequent ecological release such as in the form of increased population size or broader habitat use [36]. These measures are difficult to obtain but mechanistic frameworks that simulate these processes are currently been developed (see Wellborn and Langerhans [37]).

### What does explain the ADE-like pattern for *Pericallis*?

A key difference between *Pericallis* diversity patterns in the two archipelagos concerns the relationship between morphological and molecular (AFLP) data. We observe isolation by distance (IBD) for AFLP data in both archipelagos (Table 4). In the Canary Islands AFLP data showed some congruence with current taxonomic treatments although with sharing of genetic material evident between taxa. In the Azores, three AFLP groups were defined, broadly corresponding to the central group, São Miguel, and Santa Maria (thus two genetic SIEs are defined; Fig. 3a), a pattern that was incongruent with the recognised subspecies. These findings are similar to Schaefer et al. [10] who observed genetically differentiated SIEs in a suite of apparently widespread Azorean endemic lineages.

A smaller proportion of the AFLP variation was explained by between-island differences in the Canaries than in the Azores (Table 3), and there was greater sharing of genetic material between islands in the Canaries than between Santa Maria, São Miguel and the Central island group in the Azores. Thus, AFLP data suggest a stronger geographical signal in the Azores than in the Canaries, and the AFLP pattern in the Azores is at odds with the pattern observed with morphology whereas AFLP data and morphology are broadly congruent in the Canaries. Several factors may explain the differences. The generally smaller population sizes in the Azores than in the Canaries, partly influenced by anthropogenic factors such as habitat destruction, may have led to stronger genetic structuring. While populations in Santa Maria and, to a lesser extent, São Miguel may be large, those in the central group of the Azores are typically comprised of less than 100 individuals; in the Canaries, populations are often extensive. Geographic isolation between populations is a second factor that may explain the greater geographical structuring of AFLP data in the Azores. Colonization of a new island is the result of a combination of dispersal and establishment. *Pericallis* achenes are wind dispersed (anemochorous) that likely facilitates long distance dispersal to islands [38], yet the predominant dispersal syndromes observed in different island floras appear to be highly idiosyncratic [39]. Geographic distance is critical in the process of colonization and therefore, greater geographic distance between islands may facilitate inter-island diversification [35]. In the Canaries, the maximum distance between two neighbouring islands on which herbaceous *Pericallis* occur is ~60 km. In the Azores, the distances between Santa Maria and São Miguel (~80 km) and between São Miguel and the central group (~120 km) are both greater, and this is likely to promote greater genetic differentiation by geographic isolation (Fig. 1; [40, 41]). Within the Azorean central island group, wherein all except one accession are placed in the same genetic cluster, the islands are generally in closer proximity than in the Canaries (minimum distance: 6–19 km) and this may explain the lack of differentiation between populations on these islands. Terceira is a notable exception; at 39 km from São Jorge it is more isolated than the islands of Tenerife and La Gomera in the Canaries (28 km). The lack of differentiation of Terceira populations from other central sub-archipelago populations was also observed in genetic diversity analyses of the endemic *Picconia azorica* [42], but the island has been found to harbour distinct genetic lineages in other taxa [10].

In the Canaries, molecular and morphological diversity were both correlated with geographical distance and climatic variation (Table 4). However, geographical distance and climate were themselves correlated (r = 0.21, *P* = 0.001 for geographic distance vs PC1; r = 0.3, *P* = 0.001 for geographic distance vs. PC2). Thus, morphologically differentiated clusters in the Canarian lineage tend to be both geographically isolated and climatically differentiated (Fig. 2d). The group may therefore be considered to be an example of a classic island adaptive radiation, within which geographical isolation and ecological differentiation have acted in concert in the diversification of the group [43, 44]. In a review of molecular phylogenies of island lineages, Baldwin et al. [45] concluded that inter-island allopatry was an important driver of diversification in the Canaries given that closely related taxa often occupy apparently similar habitats but on different islands. Our results for Canarian *Pericallis* suggest that the closely related and recently diverged taxa occupy broadly similar habitats but there is some evidence for bio-climatically differentiation between taxa that may have further contributed to their diversification. Other putative examples of ‘inter-island allopatry’ in the Canaries may also involve ecological differentiation (e.g. Gonosperminae, [46]; *Lotus*, [47] or *Bystropogon* [48]).

In the Azores, morphology showed no correlation with geographical distance but was correlated with climate when the possible noise caused by geographical distance was taken into account in the partial dbRDA (Table 4). In contrast, AFLP data were not correlated with climate when geographical distance was taken into account. Thus, molecular patterns appear to reflect island isolation and genetic drift (inter-island allopatry) whereas the morphological patterns reflect ecological differentiation. The latter has involved shifts between climatic zones that have occurred within islands or island groups at least twice in parallel in the central group and in São Miguel. Therefore, in contrast to the Canaries, the effects of geographic isolation and ecological differentiation in Azorean *Pericallis* are uncorrelated.

The independent origins of the high altitude subsp. *caldeirae* ‘morphotype’ on separate islands in the central group and São Miguel may reflect underlying phenotypic plasticity, i.e. the property of a genotype to express distinct phenotypes in different environments [49]. The role of phenotypic plasticity in diversification is widely debated (see [50] and references therein). However, phenotypic plasticity provides opportunities for diversification, including ecological adaptation and speciation [50]. The maintenance of morphological differences in spite of limited genetic differentiation between taxa could also reflect strong ecological selection on few loci of large effect that are not detected by the AFLP analyses due to limited genome coverage [51–54]. Recent studies have also provided evidence for ecological divergence correlating with epigenetic changes in DNA methylation [55]. The potential role of epigenetics in generating phenotypic plasticity in the diversification of recently evolved oceanic island lineages has yet to be explored and may be significant.

Incongruence between molecular and morphological patterns may reflect a more general pattern in the Azorean flora. For example, *Euphorbia stygiana* subsp. *stygiana* shows geographical structuring of molecular data yet morphological differences to support this have not been identified [10]. Molecular studies of the Azorean *Ammi* lineage [10] and Azorean *Juniperus* [56] have demonstrated geographically structured patterns that are incongruent with morphology. It is important to note that although our sampling ensured a broad distributional range and included almost all known *Pericallis* populations in both archipelagos, the number of samples with both morphological and genetic data was limited (Additional file 4: Table S1). Therefore, future studies with an increased number of individuals per population may help further explain the patterns.

## Conclusions

Overall, our results suggest that the paucity of morphologically defined SIEs in Azorean *Pericallis* when compared to the Canaries is not simply the result of a Linnean shortfall. Furthermore, ecological diversification of taxa is observed in both archipelagos. In the Canaries, the correlation between isolation and ecological differentiation results in a classic island adaptive radiation in which we observe geographically isolated morphologically differentiated taxa, even though the molecular data suggest some gene flow. The Azorean lineage, within which morphology and molecular data are not congruent, does not conform to this pattern. The results of this study are at odds with the recent discovery of new endemic taxa in other Azorean plant lineages [29, 30, 57]. Taken together, recent work on the Azores flora suggest that its distinctiveness that was first commented on by Darwin reflects both a lack of taxonomic effort but also differences between archipelagos in the geographical and ecological context for diversification.

## Methods

### Sampling, sites and plant material

Individuals from both the Azorean (*P. malvifolia* subsp. *malvifolia* and subsp. *caldeirae*) and Canarian (*P. cruenta, P. echinata, P. murrayi, P. papyracea* and *P. steetzii*) lineages were sampled across the distribution ranges of each taxon, as recommended by Caujapé-Castells et al. [58]. In Tenerife, *P. cruenta* and *P. echinata* are known to hybridize [59]. We used morphometric analyses to identify putative hybrids. Individuals that showed intermediate morphological characteristics between the two taxa were excluded from the analysis since they were not the focus of this study. Leaf material was dried in silica gel for DNA analyses. Herbarium specimens were made and deposited at AZU, BM and ORT (Additional file 4: Table S1). Capitula were stored in 30 % alcohol for morphological analyses.

In the present study it was necessary to select samples that provided the full range of morphological characters for morphometric analysis and high quality DNA material for AFLP analyses, whilst also ensuring good geographic sampling across the distribution of taxa. In total, we sampled 150 and 114 individuals in the Azores and Canaries respectively. Some samples did not possess the morphological characters that were necessary for morphometric analyses; however, they provided high quality DNA and represented a locality that was important to sample, and they were therefore included only in the AFLP analyses. On the other hand, a number of samples possessed the full range of morphological characters for morphometric analyses yet for different reasons they could not be used for AFLP analyses. Herbarium samples, for example, typically provided poorer quality DNA than was necessary for AFLP analyses. In other cases, financial constraints limited the depth of sampling for AFLP analyses from a particular locality. Our sampling was selected to ensure a broad distributional range and to include as many known populations of *Pericallis* as possible (See Additional file 4: Table S1 for details of samples and populations). A compromise between broad geographic and taxon sampling ensuring high quality sample material whilst accounting for financial constraints was necessary for the AFLP analyses.

### Morphometric analyses

A total of 125 individuals from the Azores and 89 individuals from the Canaries were included in the morphometric analyses (Fig. 1 and Additional file 4: Table S1).

For both Canarian and Azorean lineages, the same 30 vegetative and floristic characters were initially scored (Additional file 5: Table S2 for list of morphological characters and Additional file 1: Figure S1 for illustrations of characters measured). Analyses, however, were restricted to those characters that showed some variation within lineages (Additional file 6: Table S3). Continuous characters were standardised using the function “scale” in the R v 3.0.1 package base, which transforms variables to achieve a mean of zero and a standard deviation of one [60]. Median values were taken for categorical characters that used multiple (a minimum of three) observations or counts.

FAMD, a principal component method which can assess the variation and balance the influence of both continuous and categorical variables [61], was performed in R. Missing data were accounted for using the R package missMDA and the combined (continuous and categorical) dataset was imputed using the function “imputeFAMD”. The function “FAMD” from the package FactoMineR was used on the imputed combined dataset [61]. To further test for morphological differences between taxa and between islands, the combined datasets were transformed to dissimilarity matrices for each archipelago using the “Gower” method and the function daisy in the package cluster (72, 73). Subsequently, non-parametric permutational multivariate analysis of variance (perMANOVA) were conducted using the function “adonis” from the package Vegan [62], using taxa and islands as factors, a Euclidean distance method and 999 permutations.

### Bioclimatic data

Whilst bioclimatic datasets from the WorldClim (http://www.worldclim.org/) model may not account for the complex topological variation and micro-climatic conditions and climate predictions in regions with poor station density and varied topography [63], such data has been informative in previous studies to analyse the bioclimatic characteristics of islands [35]. We, therefore, extracted values from 19 bioclimatic variables at 30 s. resolution available from Worldclim, for each collection locality using DIVA-GIS v. 7.5 (http://www.diva-gis.org/; Additional file 6: Table S3 for a list of bioclimatic variables). To assess ecological variation among localities within each archipelago, all bioclimatic variables were standardized using the same method as described for continuous morphological variables, and Principal Component Analyses (PCA) were carried out using the function “PCA” of the FactoMineR package [61]*.*

### AFLP data

The genetic markers used to date for *Pericallis* have shown very low levels of variation [18], emphasizing the need for more polymorphic genetic markers such as AFLPs. These versatile markers do not require the development of individual markers *de novo* for each species [2] and are a good choice for taxa in which little prior genomic information is available [64]. AFLPs are also an appropriate marker system when studying taxa that are polyploid, as is the case with hexaploid *Pericallis* [2, 59, 65, 66]. AFLPs have also already been used successfully to investigate hybridization between *Pericallis* taxa on Tenerife [59].

Seventy-six samples of the Azorean *P. malvifolia* were used for AFLP fingerprinting analysis, 51 of which were used in the morphometric analysis. This sampling encompassed populations of both subspecies on all islands on which they occur. Since *P. malvifolia* subsp. *caldeirae* has a more restricted distribution, only ten individuals were sampled for AFLP fingerprinting. A total of 69 samples were used for the AFLP fingerprinting analysis of the five Canarian taxa, 44 of which were used in the morphometric analysis, selected to represent the distribution range of taxa in each case (Additional file 4: Table S1).

DNA extraction followed the protocol in [18], with approximately 300 ng of genomic DNA obtained from the leaf material of each sample. Amplified fragments were obtained following the protocol of Vos et al. [67]. The restriction-ligation reaction was performed in two separate steps using the LI-COR kit (BioSciences, UK). Total genomic DNA was digested using two endonucleases: *Eco*RI-A/*Mse*I-C. Selective amplifications were carried out using fluorescent dye-labelled markers with six primer combinations. Fluorescent dye-labelled selective primers (Applied-Biosystems, Invitrogen, UK) and MyTaq™ (Bioline, UK) were used during the selective amplification phase (Additional file 7: Table S4). Polymerase Chain Reactions were conducted using a Veriti Thermal Cycler (Applied Biosystems-Invitrogen, UK). Amplified fragments were separated on an ABI 3500 Genetic Analyser at the University of Bristol using dye set DS-30 and ROX size standards (Applied Biosystems, UK). Electropherograms were scored using GeneMapper v. 3.7 (Applied Biosystems, UK). Amplified fragments of 80–500 base pairs were scored as having present (1) or absent (0) peaks in the output traces. The threshold for allele calling was set at 50 relative fluorescent units (RFU) and if a bin contained a peak above this threshold the allele was considered to be present. Before the allele frequency data were used in subsequent analyses, the results were reviewed manually using the criteria of Karudapuram & Larson [68]; we checked the size quality, genotype quality, bin assignment, allele calls and ambiguous calls.

To assess the reproducibility and reliability of AFLP fragments, we replicated 5–7 % of the individuals (6 individuals in the Canaries and 4 individuals in the Azores) at all stages from DNA isolation to AFLP production, according to the recommendations of Bonin et al. [69] and Holland et al. [70]. Duplicate analyses exhibited 85 and 90 % reproducibility of the bands for the Azores and the Canaries, respectively. We removed all loci that were not reproducible. We also removed loci and samples with >50 % missing data. The geo-referenced genotype matrices used in this paper and other relevant information can be found in the genetic diversity digests coded D-AFLP-94 and D-AFLP-97 [71, 72] in the Demiurge information system (http://www.demiurge-project.org/).

### Estimating genetic relationships and population genetic structure

Population genetic analyses of polyploids such as *Pericallis* are challenging due to the various assumptions of statistical analyses linked with the difficulty in characterising the allelic variation within each individual, and the differing inheritance patterns between loci [73]. Furthermore, it is impossible to calculate allelic frequency using AFLP data.

A Discriminant Analysis of Principal Components (DAPC) was used to assign individuals to genetic clusters [23, 24]. This is an appropriate alternative to Bayesian analysis of assignment such as STRUCTURE [74], as it does not assume Hardy-Weinberg equilibrium or make assumptions about the inheritance of each locus [73, 75]. DAPC requires the construction of prior groups, therefore we characterised the most likely number of clusters in each archipelago by running the sequential *K*-means clustering algorithm (all PCs retained) using the “find.clusters” function in the R package adegenet, based on the Bayesian Information Criterion (BIC; [24]). The analyses were run for *K* = 1–20. DAPC was then run using values of *K* around the most likely numbers of a priori clusters. The DAPC procedure consists of two steps: first, the original data are transformed and submitted to a PCA. Second, the PCs are passed to a Linear Discriminant Analysis based on the groups identified during the preliminary *K*-means clustering analysis. Retaining too many PCs with respect to the number of populations can lead to over-fitting the discriminant functions, meaning that membership probabilities may become drastically inflated for the best-fitting cluster, resulting in apparent perfect discrimination [75]. Considering this, we used the “optim.a.score” function that assesses the quality of discrimination between groups by looking at re-assignment of individuals to their prior group. The a-score can serve as a criterion for choosing the optimal number of PCs in the PCA step of DAPC [24].

For each archipelago, we used hierarchical analyses of molecular variance (AMOVA) [25, 26] to investigate partitioning of variation within and among the groups defined by the DAPC analysis. The distance between individuals was calculated from the AFLP presence/absence matrix using Dice dissimilarity index in R and the package ade4 (Dray & Dufour 2007). The Dice dissimilarity coefficient is commonly used to calculate genetic distance in polyploids [76, 77]. Significance levels were tested using 999 permutations following the procedure given by [26] and implemented in the package ade4 in R [78].

### Distance-based redundancy analyses

To investigate IBD for each archipelago, we regressed geographic distances against morphological and genetic distances by carrying out full dbRDA using the Vegan package in R [62] and calculated the significance using 1000 random permutations. Morphological distance matrices were produced following the method used for the perMANOVA analyses, using the function “daisy” and Gower’s coefficient [79] in the Cluster package in R [80]. Classic Euclidean genetic distance matrices (or Roger’s distance) were calculated from the AFLP presence/absence datasets using the “distgen.pop” function of the adegenet package [81]. Geographic distance matrices (in metres) were calculated from latitude and longitude data using the “earth.dist” function of the Fossil package [82] in R. The values were standardized using a logarithmic transformation and converted to continuous rectangular datasets using the function “npcm” of the Vegan package.

The first two PCs (PC1 and PC2) from the PCA analyses of bioclimatic variables were used as a measure of bioclimatic conditions to avoid autocorrelation between individual bioclimatic variables, and a full dbRDA was used to test for any correlation between bioclimate and morphological variation, and genetic variation as above. We used partial dbRDA to test the influence of geographical distance on the relationship between (i) genetic diversity and bioclimate and (ii) morphology and bioclimate.

## Additional files

Additional file 1: Figure S1. Factor Analyses of Mixed Data of morphological variation in the Azores, different symbols represent subspecies and islands. Each point represents an individual. (DOCX 27 kb)
Additional file 2: Figure S2. Graphs from find.clusters indicating the best *K* values for DAPC (**a**) Azores and (**b**) Canary Islands. (DOCX 265 kb)
Additional file 3: Figure S3. Discriminant Analysis of Principal Components showing the genetic clustering of populations of *Pericallis* lineages analysed in the Canaries for *K* = 3 (a) and *K* = 4 (b). Each bar represents one individual plant (69 individuals from the Canaries). (PDF 90 kb)
Additional file 4: Table S1. Sample information for accessions used in the AFLP and morphological analyses (collector’s name(s), collection number, collection date, island, locality, altitude, latitude, longitude, analysis/es applied to sample, well number (AFLP), population code for AMOVA (AFLP) and morphometric data for each sample analysed (imputed and standardized)). (XLSX 81 kb)
Additional file 5: Table S2. List of morphological traits initially scored with details of their transformations (Table A). List of morphological traits scored for (Table B) Azorean *P. malvifolia* specimens and (Table C) Canarian specimens with details of their transformations. Factor loadings of each character for the first two dimensions of the FAMD analysis are provided. Characters are sorted by values of Dimension 1. Morphological measurements referring to the list of morphological traits are indicated by figures: (Fig. A) Leaf, ray floret and disc floret measurements (refer to Table A for key and the legend); (Fig. B) Terminal peduncle; (Fig. C) Apical inflorescence bract. (DOCX 493 kb)
Additional file 6: Table S3. List of bioclimatic variables analysed and factor loadings in the (a) Azores and (b) Canary Islands. (DOCX 19 kb)
Additional file 7: Table S4. AFLP primer and labelled-dye information. (XLSX 8 kb)
