# Supplementary material for: Why do different oceanic archipelagos harbour contrasting levels of species diversity? The macaronesian endemic genus Pericallis (Asteraceae) provides insight into explaining the ‘Azores diversity Enigma’
Source: BMC Evol Biol. 2016 Oct 8;16:202. doi: 10.1186/s12862-016-0766-1 (PMC5055660; doi:10.1186/s12862-016-0766-1)
Supplement: Additional file 1: Figure S1. — Factor Analyses of Mixed Data of morphological variation in the Azores, different symbols represent subspecies and islands. Each point represents an individual. (DOCX 27 kb) [file 12862_2016_766_MOESM1_ESM.docx]

**Figure S1 Jones et al. Pericallis:** Factor Analyses of Mixed Data of morphological variation in the Azores, different symbols represent subspecies and islands. Each point represents an individual.

1. Colour coding of *Pericallis malvifolia* subsp*. caldeirae* reveals slight slight separation between individuals of ssp. *caldeirae* on different islands (ssp. caldeirae Terceira and ssp. caldeirae Faial).
2. Both subspecies (*Pericallis* subsp. *caldeirae* and subsp. *malvifolia*) on each island of occupancy are colour coded.
