# Supplementary material for: Why do different oceanic archipelagos harbour contrasting levels of species diversity? The macaronesian endemic genus Pericallis (Asteraceae) provides insight into explaining the ‘Azores diversity Enigma’
Source: BMC Evol Biol. 2016 Oct 8;16:202. doi: 10.1186/s12862-016-0766-1 (PMC5055660; doi:10.1186/s12862-016-0766-1)
Supplement: Additional file 2: Figure S2. — Graphs from find.clusters indicating the best K values for DAPC (a) Azores and (b) Canary Islands. (DOCX 265 kb) [file 12862_2016_766_MOESM2_ESM.docx]

**Figure S2 Jones et al. Pericallis** Graphs from find.clusters indicating the best *K* values for DAPC (**a**) Azores and (**b**) Canary Islands

After running the K-means clustering algorithm using the Adegenet package on the Canarian AFLP dataset using a Bayesian Information Criterion (BIC), *K*=3-5 represented the optimal number of clusters to describe the Canarian dataset (a). And *K*=3 represented the optimal number of clusters for the AFLP dataset (b).

(a)


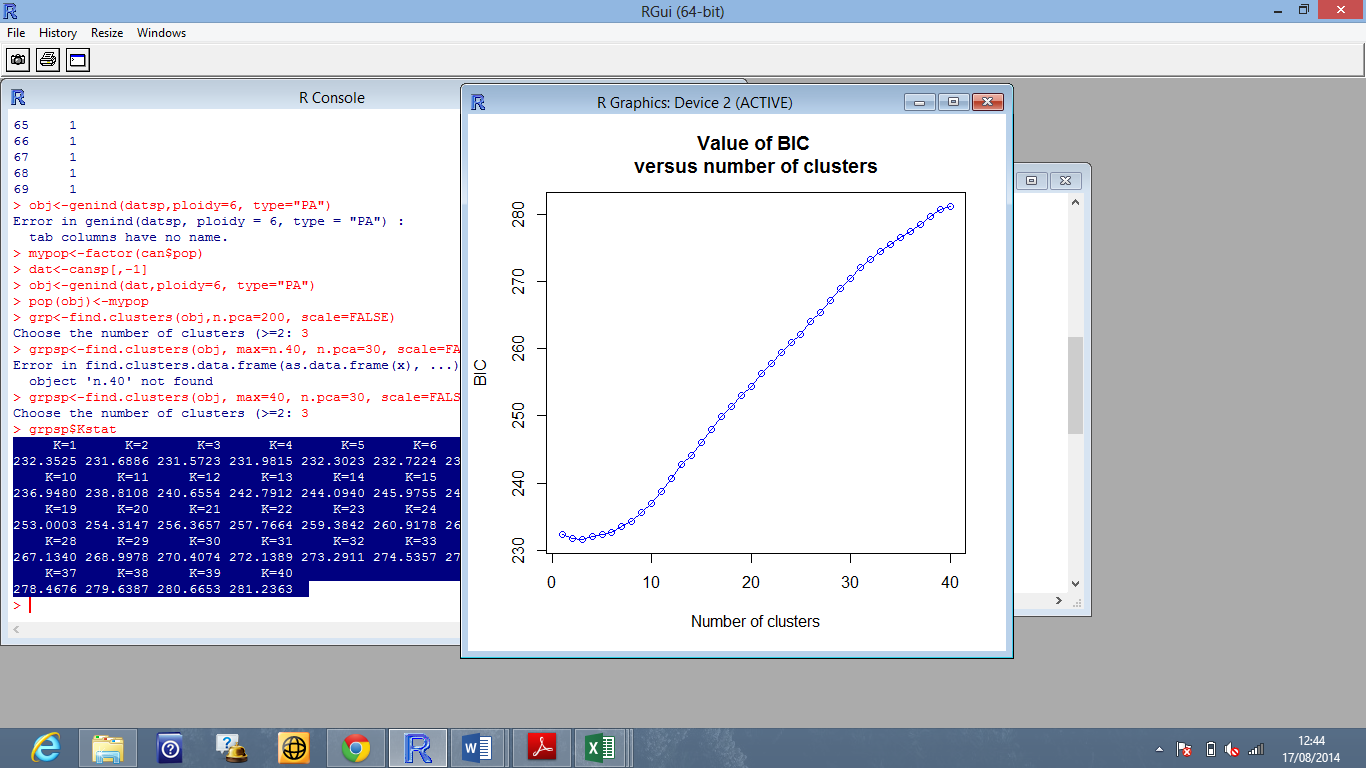


K=1 K=2 K=3 K=4 K=5 K=6 K=7 K=8 K=9

232.3525 232.2886 **231.5723** **231.5815 231.8023** 232.9224 233.5509 234.2518 235.5699

Azores


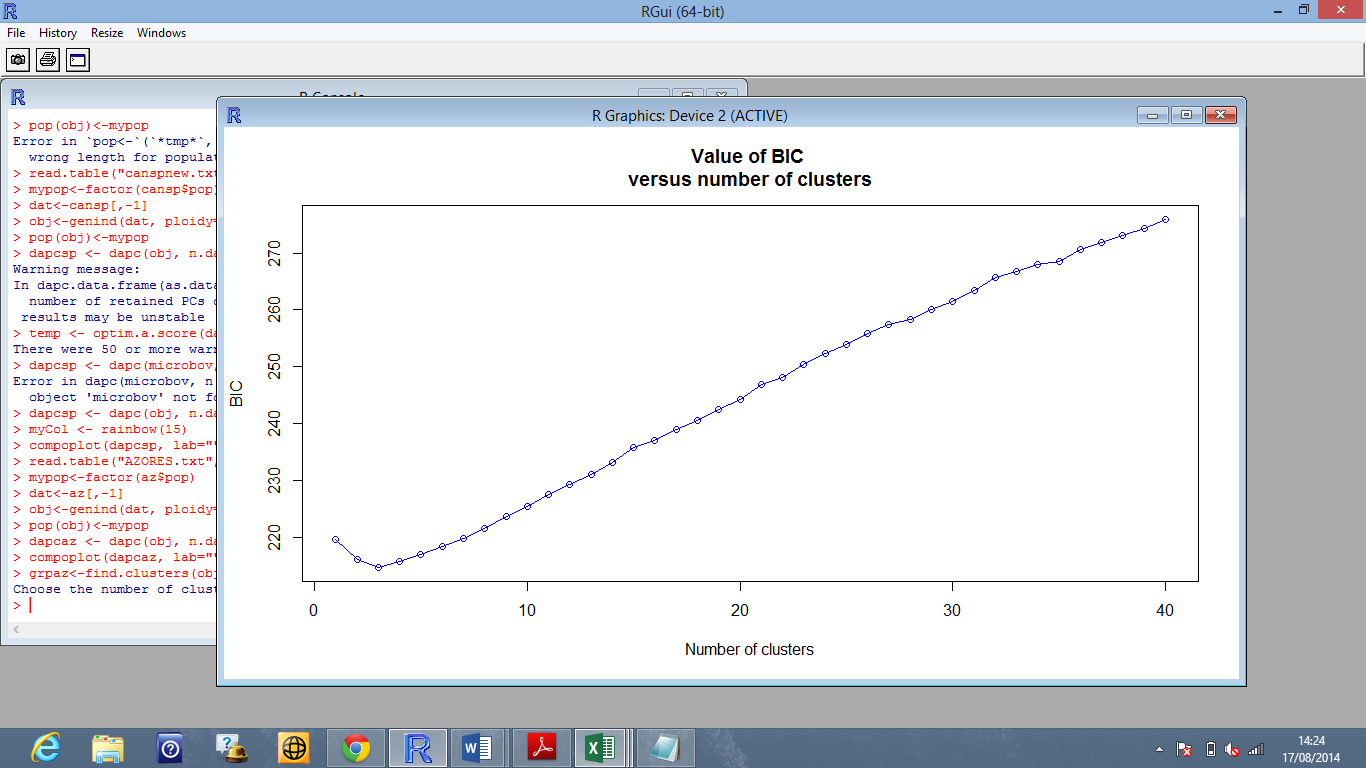


K=1 **K=2 K=3** K=4 K=5 K=6 K=7 K=8 K=9

219.5435 215.9826 **214.6858** 215.6683 216.8503 218.3626 219.7534 221.5599 223.5852
