# Supplementary figures and images for: Why do different oceanic archipelagos harbour contrasting levels of species diversity? The macaronesian endemic genus Pericallis (Asteraceae) provides insight into explaining the ‘Azores diversity Enigma’
Source: BMC Evol Biol. 2016 Oct 8;16:202. doi: 10.1186/s12862-016-0766-1 (PMC5055660; doi:10.1186/s12862-016-0766-1)

K=4

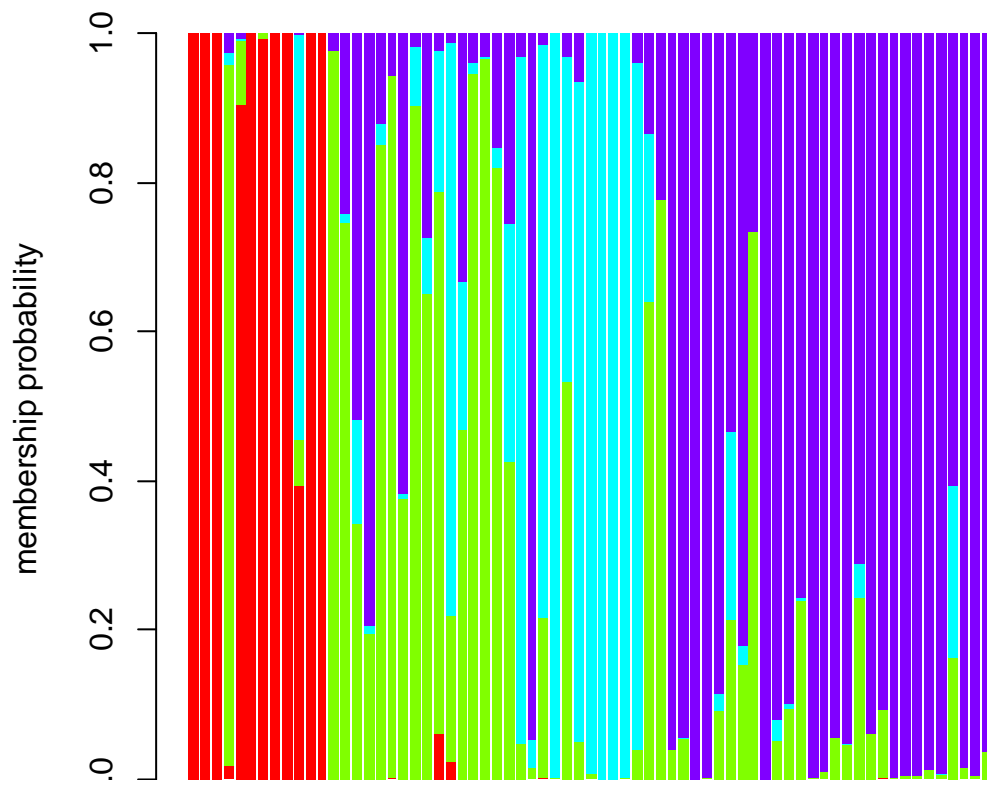

K=3

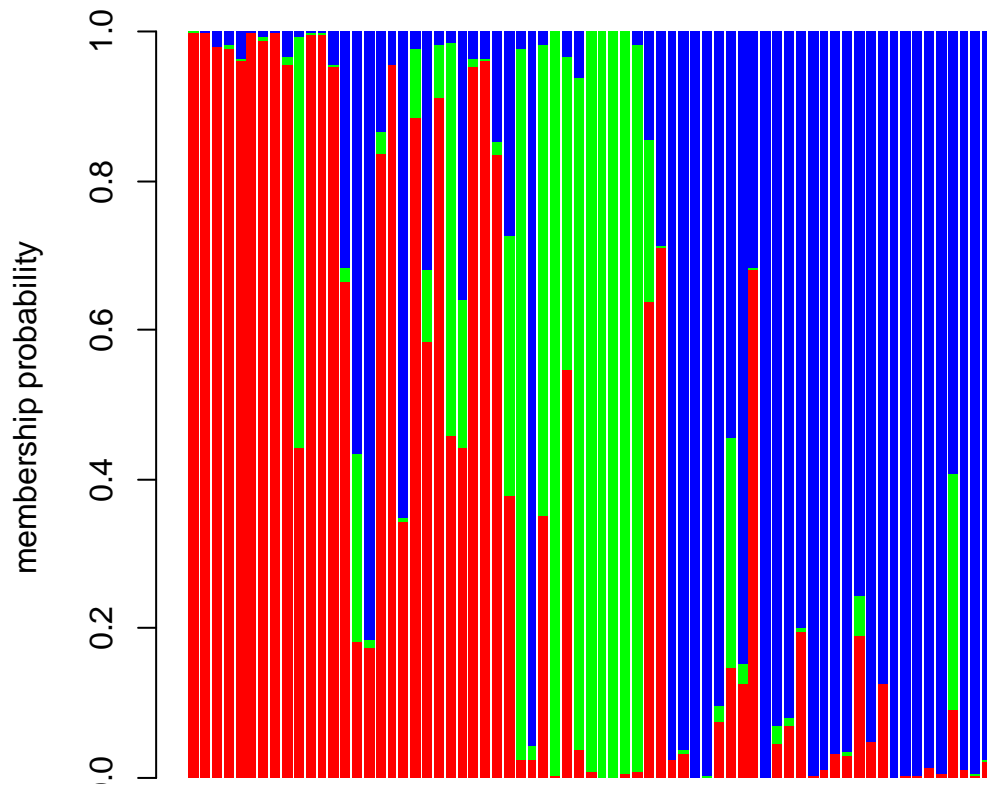

Supplement: Additional file 3: Figure S3. — Discriminant Analysis of Principal Components showing the genetic clustering of populations of Pericallis lineages analysed in the Canaries for K = 3 (a) and K = 4 (b). Each bar represents one individual plant (69 individuals from the Canaries). (PDF 90 kb) [file 12862_2016_766_MOESM3_ESM.pdf]
