# Supplementary material for: Why do different oceanic archipelagos harbour contrasting levels of species diversity? The macaronesian endemic genus Pericallis (Asteraceae) provides insight into explaining the ‘Azores diversity Enigma’
Source: BMC Evol Biol. 2016 Oct 8;16:202. doi: 10.1186/s12862-016-0766-1 (PMC5055660; doi:10.1186/s12862-016-0766-1)
Supplement: Additional file 5: Table S2. — List of morphological traits initially scored with details of their transformations (Table A). List of morphological traits scored for (Table B) Azorean P. malvifolia specimens and (Table C) Canarian specimens with details of their transformations. Factor loadings of each character for the first two dimensions of the FAMD analysis are provided. Characters are sorted by values of Dimension 1. Morphological measurements referring to the list of morphological traits are indicated by figures: (Fig. A) Leaf, ray floret and disc floret measurements (refer to Table A for key and the legend); (Fig. B) Terminal peduncle; (Fig. C) Apical inflorescence bract. (DOCX 493 kb) [file 12862_2016_766_MOESM5_ESM.docx]

| **Table S2 Jones et al. Pericallis: Table A:** List of morphological traits initially scored with details of their transformations with figures to illustrate the morphological measurements: (a) Leaf, ray floret and disc floret measurements (refer to table above for key); (b) Apical inflorescence bract; (c) Terminal peduncle. List of morphological traits scored for (Table B) Azorean *P. malvifolia* specimens and (Table C) Canarian specimens with details of their transformations. Factor loadings of each character for the first two dimensions of the FAMD analysis are provided. Characters are sorted by values of Dimension 1.  Table A |
| --- |
| **Character** |
| Stem pubescence: glabrous (0), sub-glabrous (1), hairs sparse (2), hairs dense (3) |
| Leaf length/width ratio (log) |
| Leaf length/ length to the widest point (log) |
| Leaf adaxial indumentum:glabrous (0), hairs sparse (1), hairs dense (2) |
| Leaf abaxial indumentum colour. Pink pigment: absent (0), present (1) |
| Leaf abaxial indumentum: glabrous (0), sparse (1), dense (1) |
| Lateral sinus length |
| Number of auricles: none (0), 1-3 (1), all (2) |
| Auricle length/width (log) |
| Auricle length (log) |
| Petiole length/leaf length (log) |
| Sinus width (log) |
| Petiolar sinus length (log) |
| Peduncle length (log) |
| Branchiness - number of capitula derived from branch point three branches basal from terminus. |
| Peduncle pubescence: glabrous (0) pubescent (1) |
| Length of apical inflorescence bract (log) |
| Capitula head width (at the base) (log) |
| Length of scales on the phyllary bracts (log) |
| Number of scales on the phyllary bracts (log) |
| Position of scales on the phyllary bracts: absent: (0) centre and top (1), centre and bottom (2), entire surface (3) |
| Ray floret corolla tube length (log) |
| Ray floret ligule length/width ratio (log) |
| Number of ray florets (log) |
| Ray floret ligule length/width ratio (log) |
| Ray floret colour pink pigment: absent (0), present (1) |
| Disc floret corolla tube length (log) |
| Disc floret colour. Pink pigment: absent (0), present (1) |
| Disc floret stamen length (log) |
| Disc floret cypselae indumentum: glabrous (0), spiny (1) |
| Ray stigma curvature: straight (0), slight (1), strong (2) |
| Disc floret stigma curvature: straight (0), slight (1), strong (2) |

Figures


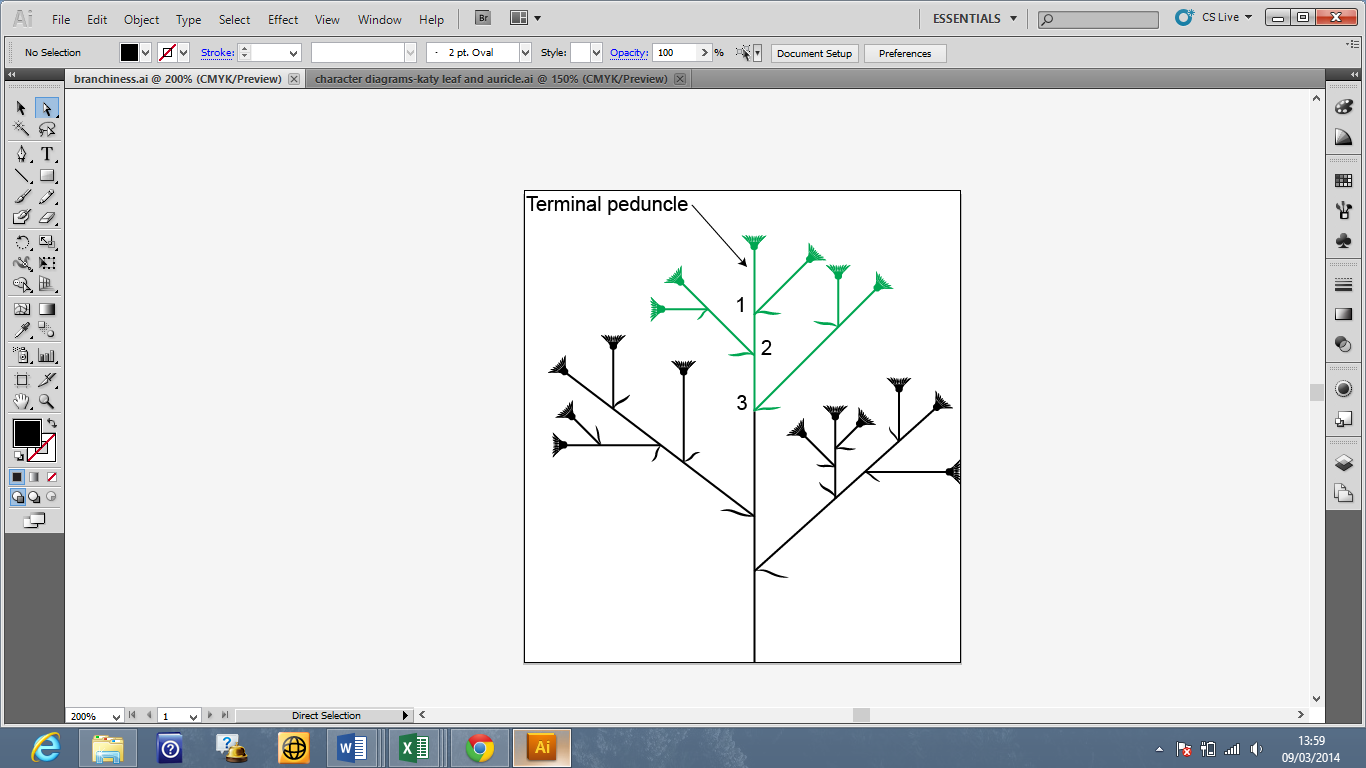

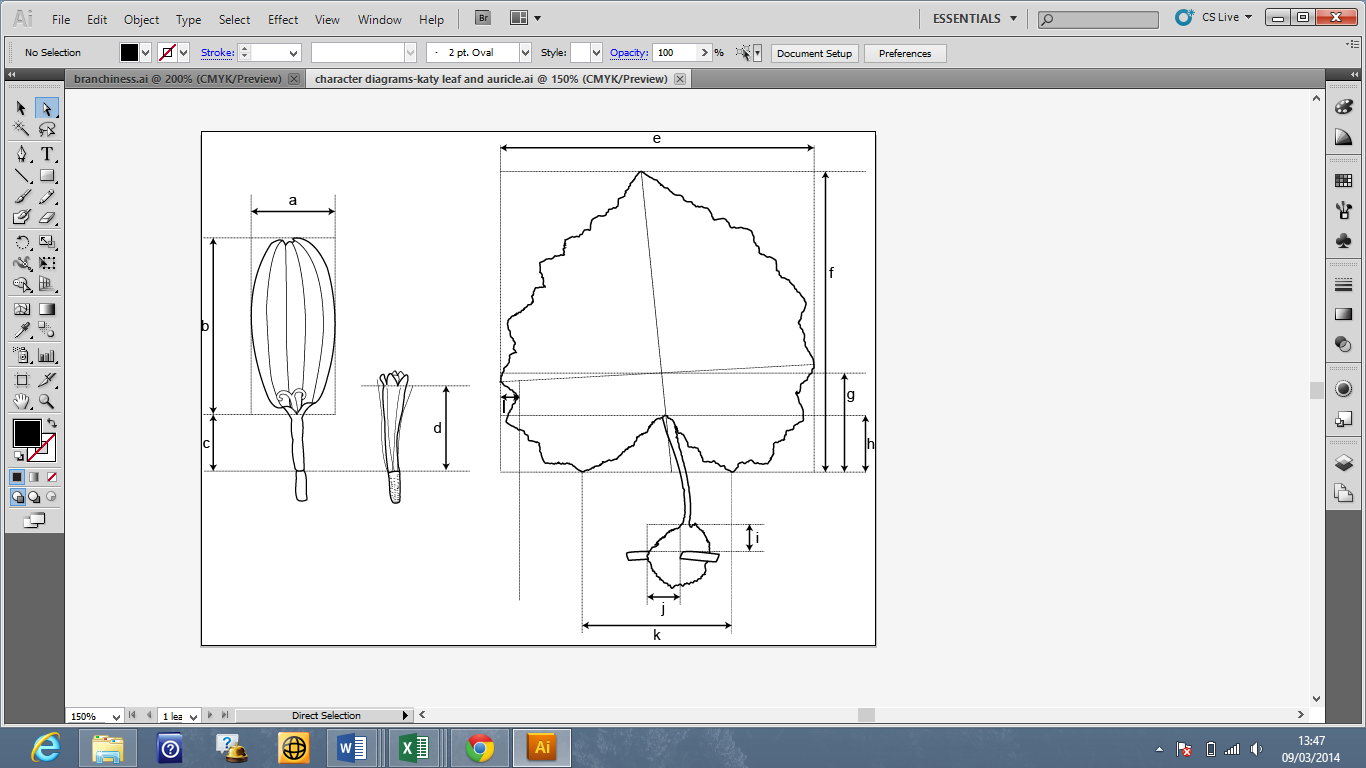


Apical

inflorescence bract

(c)

(b)

(a)

Table B

| **Trait and transformations** | **Dimension 1** | **Dimension 2** |
| --- | --- | --- |
| Length of highest bract on the peduncle (log) | 0.362 | 0.012 |
| Disc floret cypselae indumentum: glabrous (0), pubescent (1) | 0.328 | 0.094 |
| Ray floret ligule length/width ratio (log) | 0.220 | 0.130 |
| Petiolar sinus length (log) | 0.154 | 0.145 |
| Disc floret stigma curvature: straight (0), slight (1), strong (2) | 0.123 | 0.029 |
| Disc floret corolla tube length (log) | 0.106 | 0.352 |
| Number of auricles: none (0), 1-3 (1), all (2) | 0.105 | 0.093 |
| Ray stigma curvature: straight (0), slight (1), strong (2) | 0.092 | 0.035 |
| Capitula head width (log) | 0.091 | 0.196 |
| Leaf abaxial indumentum: glabrous (0), sparse (1), dense (1) | 0.026 | 0.015 |
| Leaf length/width ratio (log) | 0.021 | 0.142 |
| Disc floret stamen length (log) | 0.000 | 0.216 |

Table C

| **Trait and transformations** | **Dimension 1** | **Dimension 2** |
| --- | --- | --- |
| Capitula head width (log) | 0.290581634 | 0.017012152 |
| Ray floret corolla tube length (log) | 0.255443547 | 0.000215232 |
| Position of scales on the phyllary bracts: absent: (0) centre and top (1),  centre and bottom (2), entire surface (3) | 0.254709373 | 0.097644906 |
| Length of scales on the phyllary bract (log) | 0.250575298 | 0.005549383 |
| Peduncle pubescence: glabrous (0) pubescent (1) | 0.242509883 | 0.093358723 |
| Number of scales on the phyllary bracts (log) | 0.228851815 | 0.002189293 |
| Disc floret corolla tube length (log) | 0.201764233 | 0.005416203 |
| Peduncle length (log) | 0.191529496 | 0.053075647 |
| Number of ray florets (log) | 0.17099655 | 0.217755459 |
| Ray floret ligule length/width ratio (log) | 0.128776298 | 0.11693103 |
| Stem pubescence:  glabrous (0), sub-glabrous (1), hairs sparse (2), hairs dense (3) | 0.099098496 | 0.153915872 |
| Leaf adaxial: indumentum:glabrous (0), hairs sparse (1), hairs dense (2) | 0.070702273 | 0.232419727 |
| Disc floret colour. Pink pigment: absent (0), present (1) | 0.051256748 | 0.27692776 |
| Petiole length/leaf length (log) | 0.028504955 | 0.033010697 |
| Auricle length (log) | 0.027492456 | 0.013932566 |
| Leaf length/ length to the widest point (log) | 0.021068969 | 0.00736547 |
| Ray floret colour pink pigment: absent (0), present (1) | 0.016781484 | 0.193777382 |
| Number of auricles: none (0), 1-3 (1), all (2) | 0.012187593 | 0.007169585 |
| Leaf abaxial indumentum colour. Pink pigment: absent (0), present (1) | 0.00957684 | 0.311272853 |
| Auricle length/width (log) | 0.006528842 | 0.002085135 |
| Leaf length/width ratio (log) | 0.00471233 | 0.049210129 |
| Branchiness - number of capitula derived from  branch point three branches basal from terminus. | 0.001289296 | 0.107950639 |
| Petiolar sinus length/width (log) | 0.000281772 | 0.001522245 |
