# Supplementary material for: Why do different oceanic archipelagos harbour contrasting levels of species diversity? The macaronesian endemic genus Pericallis (Asteraceae) provides insight into explaining the ‘Azores diversity Enigma’
Source: BMC Evol Biol. 2016 Oct 8;16:202. doi: 10.1186/s12862-016-0766-1 (PMC5055660; doi:10.1186/s12862-016-0766-1)
Supplement: Additional file 6: Table S3. — List of bioclimatic variables analysed and factor loadings in the (a) Azores and (b) Canary Islands. (DOCX 19 kb) [file 12862_2016_766_MOESM6_ESM.docx]

| Bioclimatic variable | Principal component 1 | Principal component 2 |
| --- | --- | --- |
| **Precipitation of Warmest Quarter** | 0.244549744 | 0.155128523 |
| **Precipitation of Wettest Quarter** | 0.241406315 | 0.189834806 |
| **Precipitation of Wettest Month** | 0.240008895 | 0.20114288 |
| Temperature Annual Range (max. temperature of warmest month/min.temperature of coldest month) | 0.233474044 | -0.211344628 |
| Annual Precipitation | 0.232821622 | 0.234792378 |
| Precipitation of Coldest Quarter | 0.229718893 | 0.202791561 |
| Temperature Seasonality (standard deviation *100) | 0.217043511 | -0.285481523 |
| Mean Diurnal Range (Mean of monthly (max temp - min temp)) | 0.211701762 | 0.020579034 |
| *Precipitation of driest quarter* | 0.193016478 | 0.354504304 |
| *Precipitation of Driest Month* | 0.17320669 | 0.350319889 |
| Precipitation Seasonality (Coefficient of Variation) | -0.146959107 | -0.416012105 |
| Isothermality (Mean diurnal range/Temperature Annual range)x100 | -0.192609272 | 0.316852032 |
| Max. Temperature of Warmest Month | -0.248607698 | 0.131720984 |
| Min. Temperature of Coldest Month | -0.250119985 | 0.174820582 |
| Mean Temperature of Wettest Quarter | -0.250579027 | 0.172865165 |
| Mean Temperature of Coldest Quarter | -0.250638488 | 0.172618691 |
| Annual Mean Temperature | -0.251071889 | 0.165363032 |
| Mean Temperature of Warmest Quarter | -0.256717449 | 0.106402263 |
| Mean Temperature of Driest Quarter | -0.256971497 | 0.092090119 |

**Table S3** List of bioclimatic variables analysed and factor loadings in the (a) Azores and (b) Canary Islands. Bioclimatic variables extracted from World clim (<http://www.worldclim.org/>) using latitude and longitudinal values from Table S1. Factor loadings of each character for the first two principal components of the PCA analysis are given. Variables are sorted by values of Dimension 1^[[1]](#footnote-1)^ (a) in the Azores and (b) in the Canaries.

(a)

(b)

| Bioclimatic variable | Principal component 1 | Principal component 2 |
| --- | --- | --- |
| **Mean Temperature of Coldest Quarter** | 0.25703 | -0.07161 |
| **Annual Mean Temperature** | 0.255731 | -0.09642 |
| **Max Temperature of Warmest Month** | 0.254443 | -0.05038 |
| **Mean Temperature of Wettest Quarter** | 0.252886 | -0.04476 |
| **Mean Temperature of Warmest Quarter** | 0.251796 | -0.14455 |
| **Min Temperature of Coldest Month** | 0.250854 | -0.15362 |
| Precipitation Seasonality (Coefficient of Variation) | 0.19619 | 0.116597 |
| *Isothermality (Mean diurnal range/Temperature Annual range)x100* | 0.182049 | 0.451438 |
| Mean Temperature of Driest Quarter | 0.126456 | -0.14135 |
| *Mean Diurnal Range (Mean of monthly (max temp - min temp))* | 0.117003 | 0.591147 |
| *Temperature Annual Range (max. temperature of warmest month/min.temperature of coldest month)* | -0.14478 | 0.518983 |
| Temperature Seasonality (standard deviation *100) | -0.24011 | -0.20435 |
| Precipitation of Wettest Month | -0.24372 | 0.092498 |
| Precipitation of Warmest Quarter | -0.24455 | -0.0313 |
| Precipitation of Wettest Quarter | -0.24792 | 0.104586 |
| Precipitation of Coldest Quarter | -0.24963 | -0.06056 |
| Precipitation of Driest Quarter | -0.25079 | -0.11759 |
| Annual Precipitation | -0.25262 | -0.00792 |
| Precipitation of Driest Month | -0.25276 | -0.02401 |

1. Variables in bold and italics correspond to the most significant for principal component 1 and principal component 2 respectively [↑](#footnote-ref-1)
